# Restraint Stress-Induced Morphological Changes at the Blood-Brain Barrier in Adult Rats

**DOI:** 10.3389/fnmol.2015.00088

**Published:** 2016-01-14

**Authors:** Petra Sántha, Szilvia Veszelka, Zsófia Hoyk, Mária Mészáros, Fruzsina R. Walter, Andrea E. Tóth, Lóránd Kiss, András Kincses, Zita Oláh, György Seprényi, Gábor Rákhely, András Dér, Magdolna Pákáski, János Kálmán, Ágnes Kittel, Mária A. Deli

**Affiliations:** ^1^Biological Barriers Research Group, Institute of Biophysics, Biological Research Centre, Hungarian Academy of SciencesSzeged, Hungary; ^2^Biomolecular Electronics Research Group, Institute of Biophysics, Biological Research Centre, Hungarian Academy of SciencesSzeged, Hungary; ^3^Department of Psychiatry, Alzheimer's Disease Research Centre, University of SzegedSzeged, Hungary; ^4^Department of Medical Biology, University of SzegedSzeged, Hungary; ^5^Department of Biotechnology, University of SzegedSzeged, Hungary; ^6^Department of Pharmacology, Institute of Experimental Medicine, Hungarian Academy of SciencesBudapest, Hungary

**Keywords:** restraint stress, blood-brain barrier, brain endothelial cell, tight junction, glucose-transporter-1, glial endfeet, GFAP, brain capillary ultrastructure

## Abstract

Stress is well-known to contribute to the development of both neurological and psychiatric diseases. While the role of the blood-brain barrier is increasingly recognized in the development of neurodegenerative disorders, such as Alzheimer's disease, dysfunction of the blood-brain barrier has been linked to stress-related psychiatric diseases only recently. In the present study the effects of restraint stress with different duration (1, 3, and 21 days) were investigated on the morphology of the blood-brain barrier in male adult Wistar rats. Frontal cortex and hippocampus sections were immunostained for markers of brain endothelial cells (claudin-5, occluding, and glucose transporter-1) and astroglia (GFAP). Staining pattern and intensity were visualized by confocal microscopy and evaluated by several types of image analysis. The ultrastructure of brain capillaries was investigated by electron microscopy. Morphological changes and intensity alterations in brain endothelial tight junction proteins claudin-5 and occludin were induced by stress. Following restraint stress significant increases in the fluorescence intensity of glucose transporter-1 were detected in brain endothelial cells in the frontal cortex and hippocampus. Significant reductions in GFAP fluorescence intensity were observed in the frontal cortex in all stress groups. As observed by electron microscopy, 1-day acute stress induced morphological changes indicating damage in capillary endothelial cells in both brain regions. After 21 days of stress thicker and irregular capillary basal membranes in the hippocampus and edema in astrocytes in both regions were seen. These findings indicate that stress exerts time-dependent changes in the staining pattern of tight junction proteins occludin, claudin-5, and glucose transporter-1 at the level of brain capillaries and in the ultrastructure of brain endothelial cells and astroglial endfeet, which may contribute to neurodegenerative processes, cognitive and behavioral dysfunctions.

## Introduction

Most stress episodes provoke a controlled response of the organism, which is essential for survival and may enhance performance. Intense or prolonged exposure to stress, in contrast, may lead to a variety of neuropsychiatric disorders (Chrousos, 2009). Stressful stimuli are reported to impair synaptic plasticity in the hippocampus and to inhibit hippocampal neurogenesis (Kim et al., 2013; Maggio et al., 2013; Schoenfeld and Gould, 2013; Grigoryan et al., 2014). Furthermore, retrospective epidemiological studies indicate that stress is associated with increased risk of dementia and development of neurological or psychiatric disorders, such as Alzheimer's disease or major depressive disorder (Wilson et al., 2006, 2007; Najjar et al., 2013).

The effect of stress is influenced by a number of factors, including individual sensitivity, the brain region affected and the type of stress (Chen et al., 2012). Experimental animal models should reproduce the pathophysiological features of human diseases. One of the commonly used models is restraint stress, which is a modified form of immobilization stress. During this procedure inescapable physical and mental stress is induced by placing the animals in a plastic tube in order to block their movements. This is a validated experimental stressor involving both physical and psychological effects at the same time (Pitman et al., 1988; Jaggi et al., 2011). Frontal cortex and hippocampus are brain regions sensitive to stress-induced damage (Kim and Diamond, 2002; Radley et al., 2006). Chronic (8-week) restraint stress leads to learning and memory impairment, and neuronal damage in the frontal cortex and the CA1 region of the hippocampus in rats (Huang et al., 2015). Recently, we showed that acute and chronic restraint stress in rats provokes alterations in gene and protein expression of β-actin, the major cytoskeletal component of dendritic spines, and of amyloid precursor protein, which is associated with Alzheimer's disease in hippocampus but not in frontal cortex (Sántha et al., 2012). The present work is a follow up morphological study of the publication of Sántha et al. using the same restraint-induced stress model in rats focusing on BBB changes and examining possible neuronal damage in the same two brain regions.

Stress stimuli, among other challenges emerging from the external and internal environment, alter circulating plasma composition. The central nervous system (CNS) is protected from these fluctuations by barriers, among which the blood-brain barrier (BBB) plays a key role in maintaining homeostasis. The BBB supplies the brain with oxygen, glucose, and other nutrients required for neural functions, it contributes to the optimal ionic and transmitter composition of the neural microenvironment for synaptic signaling, and protects the CNS against neurotoxic substances (Abbott et al., 2010).

The BBB is formed by non-fenestrated endothelial cells that line cerebral microvessels. These specialized endothelial cells contact each other with tight junctions (TJs). TJs form an effective physical barrier against the diffusion of hydrophilic solutes via the paracellular pathway. Consequently, water-soluble substances, including glucose, cross the BBB by transcellular mechanisms using specific carriers. The transport of glucose through the BBB by glucose transporter-1 (GLUT-1) is crucial, because glucose is the primary energy source of the brain (Leybaert et al., 2007). The effectiveness of the barrier function especially depends on the expression and function of the main structural proteins of TJs, such as occludin, claudin-3, and claudin-5 (Abbott et al., 2010). Brain capillary endothelial cells are surrounded by pericytes, and the microvessel wall is ensheathed by astroglial endfeet (Abbott et al., 2006). The contribution of astrocytes in barrier induction and maintenance is considered fundamental (Abbott, 2002). Besides producing and releasing inductive signals that regulate the BBB phenotype (Haseloff et al., 2005), astrocytes take part in maintaining the ionic, water, amino acid, and neurotransmiter homeostasis of the CNS, and provide the cellular link to neurons (Abbott et al., 2006).

The BBB is a highly dynamic structure. Agents released during neural activity like histamine, glutamate, leptin, Ca^2+^, adrenaline, serotonin act on receptors and transporters expressed on endothelial cells and astrocytes, thus modulate capillary diameter, local blood flow (Peppiatt et al., 2006; Dalkara and Alarcon-Martinez, 2015), GLUT-1 expression (Boado and Pardridge, 2002), tightness of TJs, and other features of the BBB phenotype (Huber et al., 2001; Zlokovic, 2008). BBB functions are coupled to and regulated by local neural activity in physiological circumstances (Neuwelt et al., 2011). In contrast, under various pathological conditions, including systemic or CNS diseases, serious perturbations of BBB functions are reported (Zlokovic, 2011; Winkler et al., 2015). While the role of the BBB is increasingly recognized in the development of neurodegenerative disorders, such as Alzheimer's disease (Erickson and Banks, 2013), dysfunction of the BBB has been linked to stress-related psychiatric diseases only recently. Psychiatric disorders in which blood-brain barrier damage/dysfunction is implicated include major depressive disorder (Najjar et al., 2013), neuropsychiatric lupus (Meszaros et al., 2012), cognitive impairment caused by obstructive sleep apnea (Lim and Pack, 2014), and schizophrenia (De Klerk et al., 2011; Bechter, 2013). In the majority of these diseases stress is a pathogenic factor and contributes to the progression of neuroinflammation and neuronal death. Stress stimuli related to the pathomechanism of CNS diseases, including Alzheimer's disease (Jeong et al., 2006; Grigoryan et al., 2014), are also reported to directly influence BBB functions (Foti Cuzzola et al., 2013).

However, there are few studies on immobilization stress related changes at the BBB, which focus on increased BBB permeability (Skultétyová et al., 1998) and alterations in brain catecholamines and serotonin (Belova and Jonsson, 1982; Esposito et al., 2002; Shah et al., 2003). The impact of immobilization stress on the cellular and molecular components of the BBB is still unexplored.

The present study aims to provide a complex analysis of BBB morphological changes during acute and chronic restraint stress in two stress sensitive areas of the rat brain, the hippocampus and the frontal cortex. Image analysis of the immunoreactivity of proteins characterizing brain endothelial cells and astrocytes was carried out to reveal stress related alterations in their fluorescence intensity, suggesting quantitative changes. The entropy and complexity of the immunostaining pattern of two key TJ structural proteins, occludin, and claudin-5 was studied in order to highlight rearrangements in their localization. Furthermore, electron microscopic analysis of brain microvessels in stressed animals was performed to examine the ultrastructure of brain endothelial cells and astrocytes, the two basic components of the BBB. In order to examine whether restraint stress leads to neuronal damage in this model, density of NeuN immunoreactive neurons was investigated in both stress sensitive brain regions.

## Materials and methods

### Materials

All reagents were purchased from Sigma-Aldrich Ltd. (St. Louis, MO, USA) except for those specifically mentioned.

### Animals

The experiments were performed on 17 adult male Wistar rats (~350 g), housed under standard laboratory conditions with a 12 h light/dark cycle, at constant temperature (22 ± 1°C) and humidity (55 ± 5%) in the conventional animal house of the Biological Research Centre, Szeged, Hungary. Free access was allowed to tap water and standard rodent chow. All experimental procedures were approved by the 1998. XXVIII. Hungarian law and the EU Directive 2010/63/EU about animal protection and welfare. Formal approvals for animal studies have been obtained from the local Hungarian animal health authority, the Governmental Office for Csongrád County, Directorate of Food Chain Safety and Animal Health (Permit number: XVI./834/2012).

### Stress procedure

Restraint stress was performed (5 h/day for 1, 3, and 21 days) as described previously (Sántha et al., 2012). For the stress procedure animals were randomly divided into four experimental groups. Group 1 (*n* = 4) contained control animals that were left completely undisturbed, while group 2 (*n* = 4), group 3 (*n* = 4), and group 4 (*n* = 4) included rats receiving restraint stress for 1, 3, and 21 days, respectively. The body weight of the animals, as a validated stress marker, was measured on the days of perfusion. The control group represented a pair-fed group kept in the same housing and feeding conditions.

### Immunohistochemistry

The day after the last stress procedure, rats were anesthetized with Avertin [2% 2,2,2-tribromoethanol (T48402), 8% ethanol (E7023), 1.2 % 2-methyl 2 buthanol (240486); 1 ml/100 g body weight]. The animals were perfused transcardially with cold saline solution (0.9% NaCl, 746398) containing heparin (H3393, 100 U/ml, 200–250 ml/animal). Brains were fixed with 3% paraformaldehyde (158127) in phosphate buffered saline (PBS, 0.1 M, pH 7.4), then cryoprotected with increasing concentrations of sucrose (1623637), solutions (10–20–30% sucrose in PBS) on three consecutive days) and stored in 30% sucrose-PBS at 4°C until sectioning. The frontal brain region (Bregma 5.2–2.7 mm) and the midbrain region (Bregma 1.8–6.3 mm) were cut into 15-μm-thick sagittal sections on a cryostat (Floorstanding Cryostat MNT; Slee, Mainz, Germany) and the slices were kept in 0.1% azide-PBS solution at 4°C until performing immunohistochemical stainings. Free-floating sections were washed in PBS, then an antigen retrieval step using 10 mM citrate buffer [1 mM citric acid (C1909), 10 mM sodium citrate (S4641), pH 6] for 20 min at 70°C was carried out for GLUT-1 and GFAP immunostainings, then sections were incubated in 0.5% Triton X-100 (T8787) in PBS for 30 min. In case of claudin-5 and occludin sections were incubated in 0.5% Triton X-100 in PBS for 30 min which was followed by treatment with 10 μg/ml pronase (Protease Type XIV, P5147) in CaCl_2_ (223506) solution for 7 min. Unspecific binding sites were blocked with 1% bovine serum albumin (A9418) and 2% fetal bovine serum (P40-1301, Pan Biotech, Aidenbach, Germany) in PBS, then sections were incubated overnight with the following primary antibodies at 4°C: anti-GFAP (mouse monoclonal antibody, G3893, 1:400), anti-GLUT-1 (rabbit polyclonal antibody, SAB4502803, 1:200), anti-claudin-5 (rabbit polyclonal antibody, SAB4502981, 1:200), anti-occludin (rabbit polyclonal antibody, 71–1500, Thermo Fisher Scientific, Waltham, MA, USA; 1:100). The next day, after three washes with PBS the samples were incubated with the corresponding secondary antibodies: Alexa Fluor-488-labeled anti-mouse IgG (A-11029, Thermo Fisher Scientific, 1:400) and Cy3-labeled anti-rabbit IgG (C2306, 1:1000) for 1 h. After this incubation the sections were washed five times for 5 min with PBS, then cell nuclei were counterstained with Hoechst dye 33342 (PA-3014, Lonza, Walkersville, MD, USA; 6 μg/ml). The samples were mounted in Fluoromount-G (0100-01, Southern Biotech, Birmingham, AL, USA) and were sealed with CoverGrip Coverslip Sealant (PI-23005, Biotium Inc., Hayward, CA, USA). Specificity of the staining was checked by incubating the sections with secondary antibodies only, and no background stainings were found. The immunostained sections were examined with Leica SP5 (Leica Microsystems GmbH, Wetzlar, Germany) confocal laser scanning microscope using a 63x objective lens with 3x zoom factor with sequential scan procedure. Each immunostaining pattern was analyzed using stained sections from both regions (frontal cortex, hippocampus) from each animal. Non-overlapping digital images (512 × 512 pixel, *n* = 6–14) were taken from both the frontal cortex and hippocampus with standardized parameter settings and all images were analyzed using ImageJ (National Institutes of Health, Bethesda, MD, USA), ImageQuant™ (Molecular Dynamics GmbH, Krefeld, Germany) and MATLAB (Mathworks, Natick, MA, USA) softwares.

In the case of NeuN immunostaining NeuN expressing neurons were visualized with the avidin-biotin-peroxidase method (Hsu et al., 1981). Endogenous peroxidase activity was blocked by incubating the free-floating sections in 3% H_2_O_2_ and 20% methanol in PBS for 15 min, while non-specific immunoreactivity was blocked by incubating the sections in 5% normal horse serum (H0146) and 0.3% bovine serum albumin in PBS for 2 h at room temperature. Then, sections were incubated in anti-NeuN primary antibody (mouse monoclonal antibody, MAB377, Merck Millipore, Darmstadt, Germany, 1:1000) at room temperature overnight. Following incubation with the primary antibody sections were rinsed in PBS (3 × 10 min) and incubated with biotinylated donkey anti-mouse IgG secondary antibody (715-065-150, Jackson ImmunoResearch Ltd., Newmarket, UK, 1:1000) for 2 h at room temperature. Then, sections were washed in PBS (3 × 10 min) and incubated in Vectastain Elite ABC reagent (PK-6100, Vector Laboratories, Burlingame, CA, USA, 1: 250) for 2 h at room temperature. Sections were rinsed in PBS, then the reaction product was visualized by incubating the sections in 0.042% 3,3′-diaminobenzidine (D3939) and 0.002% hydrogen peroxide (H3410) in PBS, pH 7.4 for 5 min. The immunostained sections were mounted on slides, dehydrated in an ascending series of alcohol and coverslipped with Entellan (107960, Merck, Darmstadt, Germany). Non-overlapping digital images were taken with an Olympus BH2 light microscope (Olympus, Tokyo, Japan).

### Image analysis

For semiquantitative analysis, the color pictures were converted to grayscale 8-bit TIFF file format with Adobe Photoshop software (Adobe Systems Inc., San Jose, CA, USA) as described previously (Farkas et al., 2008). Fluorescence intensity of images was evaluated using the ImageQuant™ software as follows: on every image 10 equally sized small rectangular areas (7 × 7 pixels) were placed randomly on the intensively highlighted immunolabeled structures, and five equal rectangles were placed randomly on areas lacking immunostaining representing the background. Then the average intensity/pixel values of each area were calculated, and the average intensity/pixel values representing the background intensity were subtracted from those of immunolabeled areas. This procedure was performed on each image then the collected data were statistically analyzed (Farkas et al., 2008). The complexity of the TJ proteins was quantitatively analyzed with the public domain ImageJ software. After measuring the length and branch points of TJs the complexity index of TJs was calculated as the ratio of the number of branch points and the length of TJ strands (Kniesel and Wolburg, 1993). The orderliness of the staining pattern was studied with analysis of the mean object size and the entropy of the images using the MATLAB software. The ratio of the summarized object size and the number of objects determined the mean object size. The entropy was calculated according to the information theory of entropy based on the Markov model. For the implementation of the method, the images were converted to grayscale, and then the MATLAB functions “entropy” and “imhist” were used. “Imhist” provides the histogram of the image (where the default value of 256 was used as the number of bins), while the “entropy” command calculates the entropy of the image according to the formula—sum(p.^*^log_2_p), where *p* contains the histogram values returned from “imhist” (Gonzalez et al., 2003). For all four analyses data of the control group were regarded as 100% and the data of groups 2–4 were compared to those of the control group.

To determine the density of NeuN expressing neurons, images were analyzed with the public domain Image J software. First, pictures were converted to grayscale 8-bit images and the area of interest in the frontal cortex, CA1 region of the hippocampus and polymorphic region of the hippocampal dentate gyrus was measured. Then, grayscale 8-bit images were converted to thresholded 1-bit images, on which the number of immunolabeled structures localized in the area of interest was measured considering particles larger than 50 μm^2^ and with circularity values between 0 and 1. Last, the number of NeuN immunoreactive cells per 1000 μm^2^ was calculated.

### Electron microscopy

Rats were anesthetized with Avertin (1 mg/ml body weight) and then briefly perfused through the ascending aorta with 0.9% NaCl solution containing 100 U heparin, followed by 3% paraformaldehyde containing 0.5% glutaraldehyde (G5882) in cacodylate buffer [0.05 M cacodylate (C4945), 0.25 M sucrose (1623637), in 0.9% NaCl, pH 7.4, ~1 ml/g body weight] for 30 min. Brains were removed and left for a 24 h postfixation period in the same buffer at 4°C. After postfixation, samples were washed with the fixative several times and 40 μm thick coronal sections were cut on an Oxford Vibratome (The Vibratome Company, St. Louis, MO, USA). Sections were washed with PBS and post-fixed in 1% OsO_4_ (75633) for 30 min then rinsed with distilled water and dehydrated in graded ethanol, block-stained with 1% uranyl acetate in 50% ethanol for 30 min, and embedded in Taab 812 (T004, Taab; Aldermaston, UK). Following polymerization at 60°C for 12 h, 60–70 nm ultrathin sections were cut using a Leica UCT ultramicrotome (Leica Microsystems, Milton Keynes, UK) and examined using a Hitachi 7100 transmission electron microscope (Hitachi Ltd., Tokyo, Japan). Electron micrographs were made by Veleta 2k × 2k MegaPixel side-mounted TEM CCD camera (Olympus). Contrast/brightness of electron micrographs was edited by Adobe Photoshop CS3 (Adobe Photoshop Inc., San Jose, CA, USA). Altogether 264 non-overlapping images representing 57 capillaries from the frontal cortex and 269 non-overlapping images representing 58 capillaries from the hippocampus were analyzed for morphological changes. All analyzed images were taken at 30,000X magnification.

### Statistical analysis

GraphPad Prism 5.0 software (GraphPad Software Inc., La Jolla, CA, USA) was used for statistical analysis. Gaussian distribution of the data was tested with the KS normality test. Data showing Gaussian distribution were analyzed with One-way ANOVA followed by Bonferroni *post hoc* test. Data showing no Gaussian distribution were analyzed with One-way ANOVA followed by Kruskal–Wallis and Dunn's multiple comparison tests. The level of statistical significance was taken as *p* < 0.05. Results are presented as means ± SEM.

## Results

### Change in body weight gain as a marker of stress response

**Figure 1:**
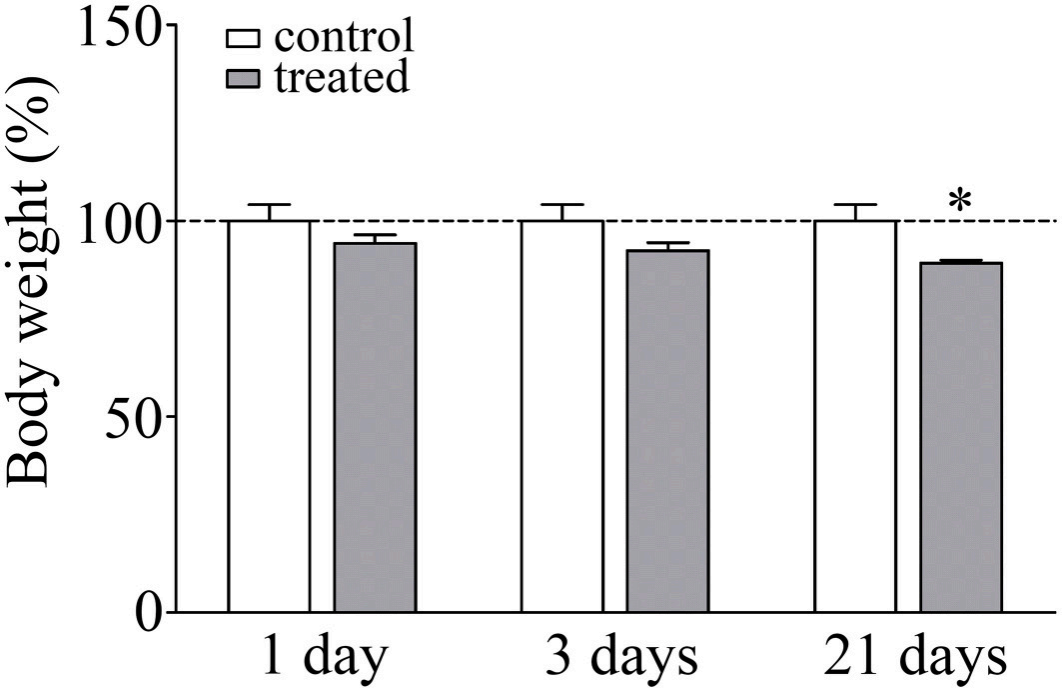
**Effect of restraint stress on the body weight of rats**. Results are expressed as percentages of the unstressed control group. Values are group means ± SEM, *n* = 4–5. Data were analyzed by One-way ANOVA followed by a Bonferroni *post hoc* test. ^*^p < 0.05, significant differences as compared to the control.

The body weight of the animals was monitored during the experiments as a read-out of stress response. There was a significant lack of gain in body weight in response to 21 days of restraint stress (92.4%; *p* < 0.05) compared to the unstressed pair-fed control rats (Figure 1). The significant decrease in body weight gain is a pathophysiological indicator of stress and a stress parameter generally accepted in the literature (Chiba et al., 2012; Sántha et al., 2012; Filaretova et al., 2013).

### Immunohistochemical analysis of brain capillary endothelial cells

#### Immunostaining pattern of tight junction proteins claudin-5 and occludin

**Figure 2:**
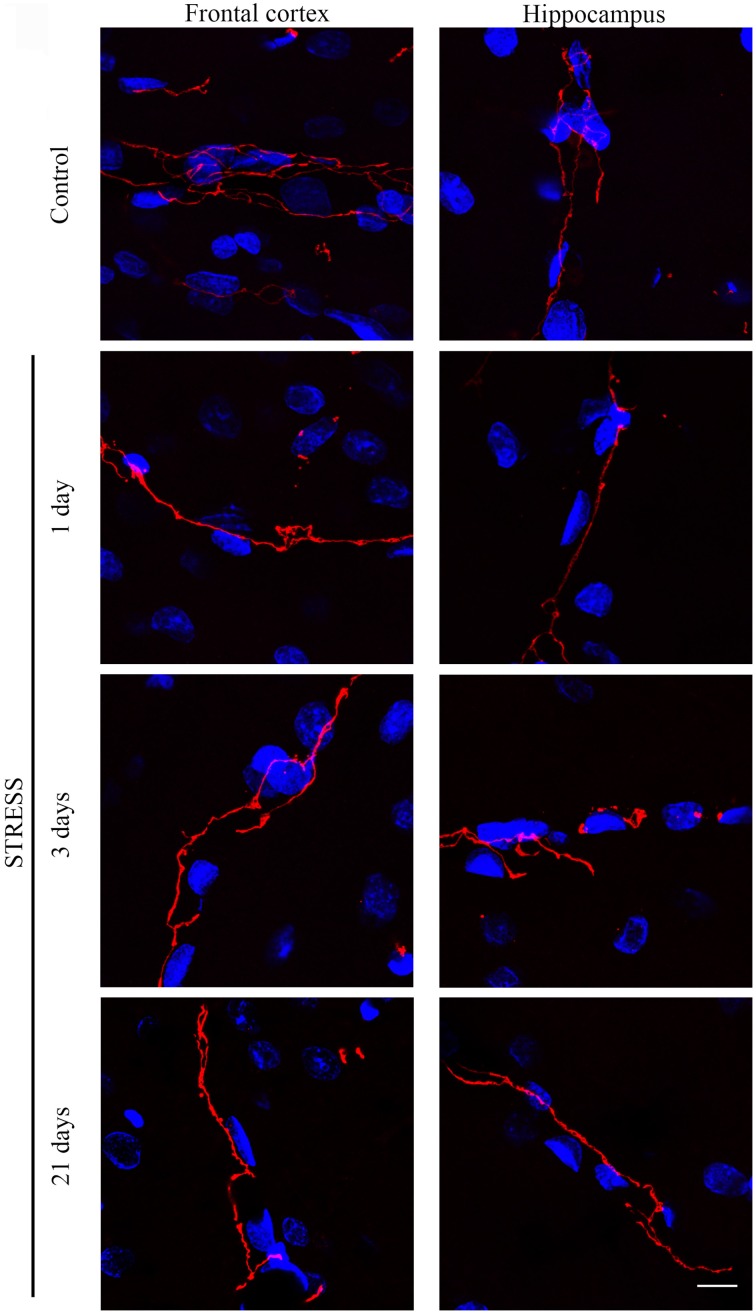
**Expression of claudin-5 in rat frontal cortex and hippocampus of control and stressed rats**. Sagittal brain sections of control and stressed rats were immunostained for tight junction protein claudin-5 of brain capillaries. Red color: immunostaining for claudin-5. Blue color: cell nuclei stained with Hoechst dye 33342. Scale bar: 15 μm.

Stress-induced morphological changes on brain endothelial TJs were examined by anti-claudin-5 and occludin immunostainings which only marked this cell type in the examined brain regions. The immunohistochemical detection of both proteins revealed a complex, branched network of TJ structrural proteins in the hippocampus and frontal cortex of control animals. In contrast, in brain capillaries of rats receiving restraint stress claudin-5 and occludin showed an altered morphology based on their immunostaining pattern (Figures 2, 4).

**Figure 3:**
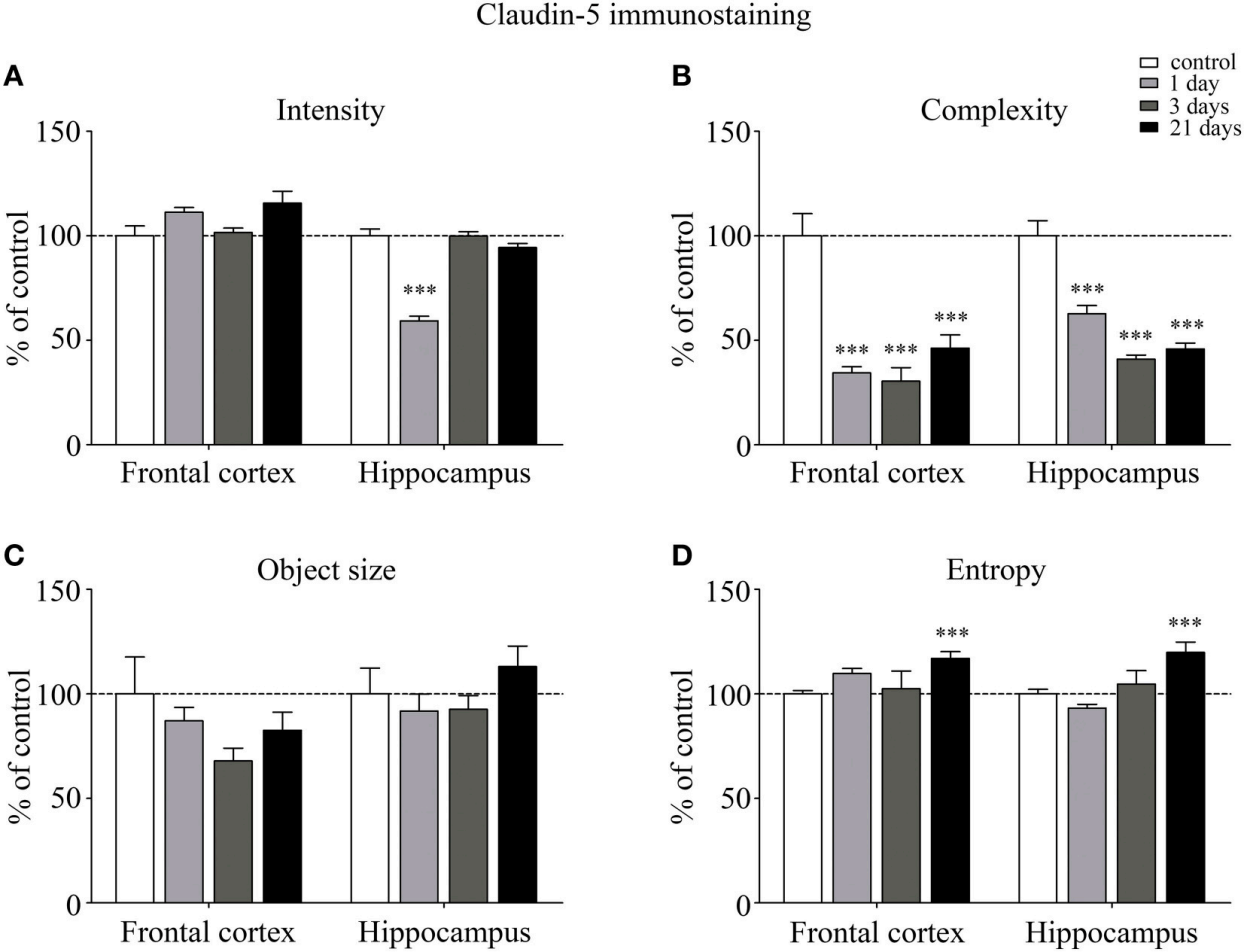
**Analysis of claudin-5 immunofluorescence pattern in the frontal cortex and hippocampus of rats following restraint stress**. Graphs represent quantification of the intensity **(A)**, complexity **(B)**, mean object size **(C)**, and entropy **(D)** of immunostained structures. Results are expressed as percentages of the unstressed control group. Values for each group are means ± SEM. Data were analyzed by One-way ANOVA followed by a Bonferroni *post hoc* test. ^***^p < 0.001, significant differences as compared to the unstressed control group.

The fluorescence intensity of integral membrane TJ protein claudin-5 was significantly reduced in the hippocampus of rats after acute, 1 day restraint stress (Figure 3A). The complexity of claudin-5 immunostaining pattern decreased in both the frontal cortex and hippocampus in all stress groups (*p* < 0.001; Figure 3B). The discontinuity of the staining was especially remarkable in the hippocampus after 3 days of stress. No significant change was observed regarding the mean object size neither in the frontal cortex nor in the hippocampus (Figure 3C) at any time point tested. The entropy of the claudin-5 staining was significantly elevated in both the frontal cortex and hippocampus on day 21 of restraint stress (*p* < 0.001; Figure 3D).

**Figure 4:**
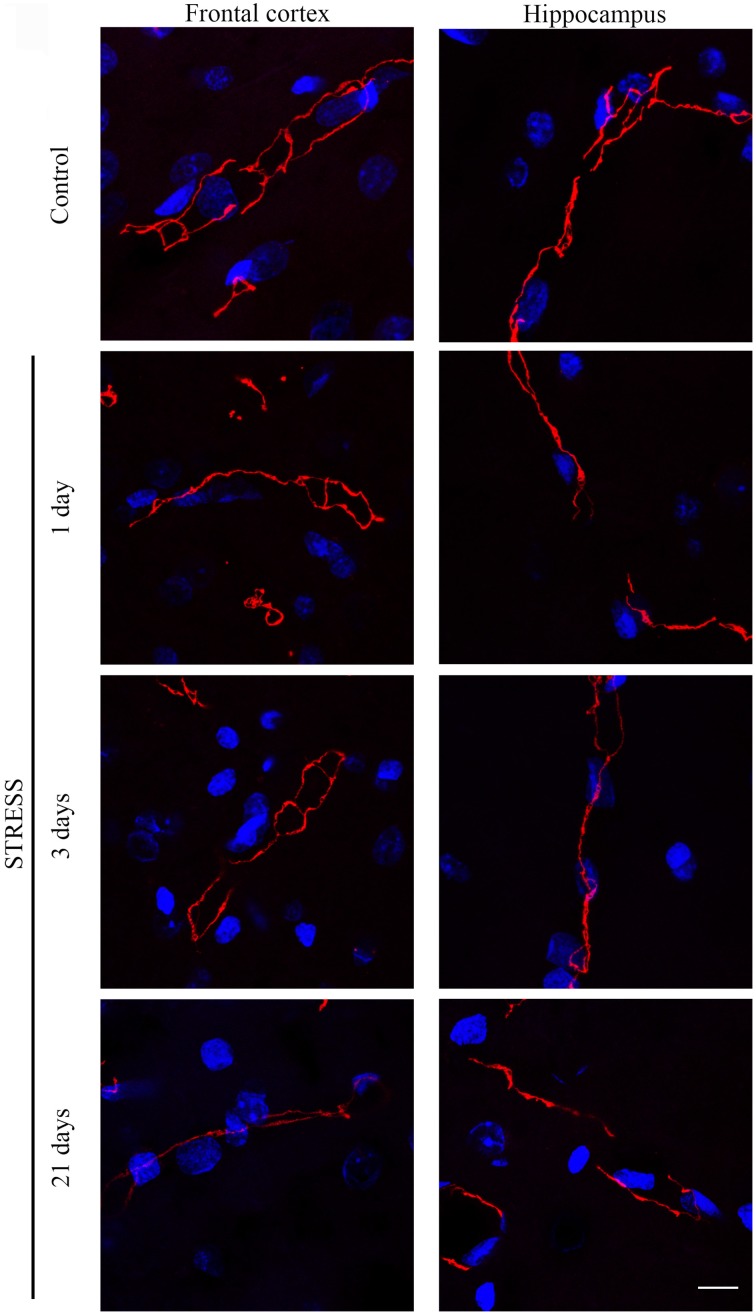
**Expression of occludin in rat frontal cortex and hippocampus of control and stressed rats**. Sagittal brain sections of control and stressed rats were immunostained for tight junction protein occludin of brain capillaries. Red color: immunostaining for occludin. Blue color: cell nuclei stained with Hoechst dye 33342. Scale bar: 15 μm.

**Figure 5:**
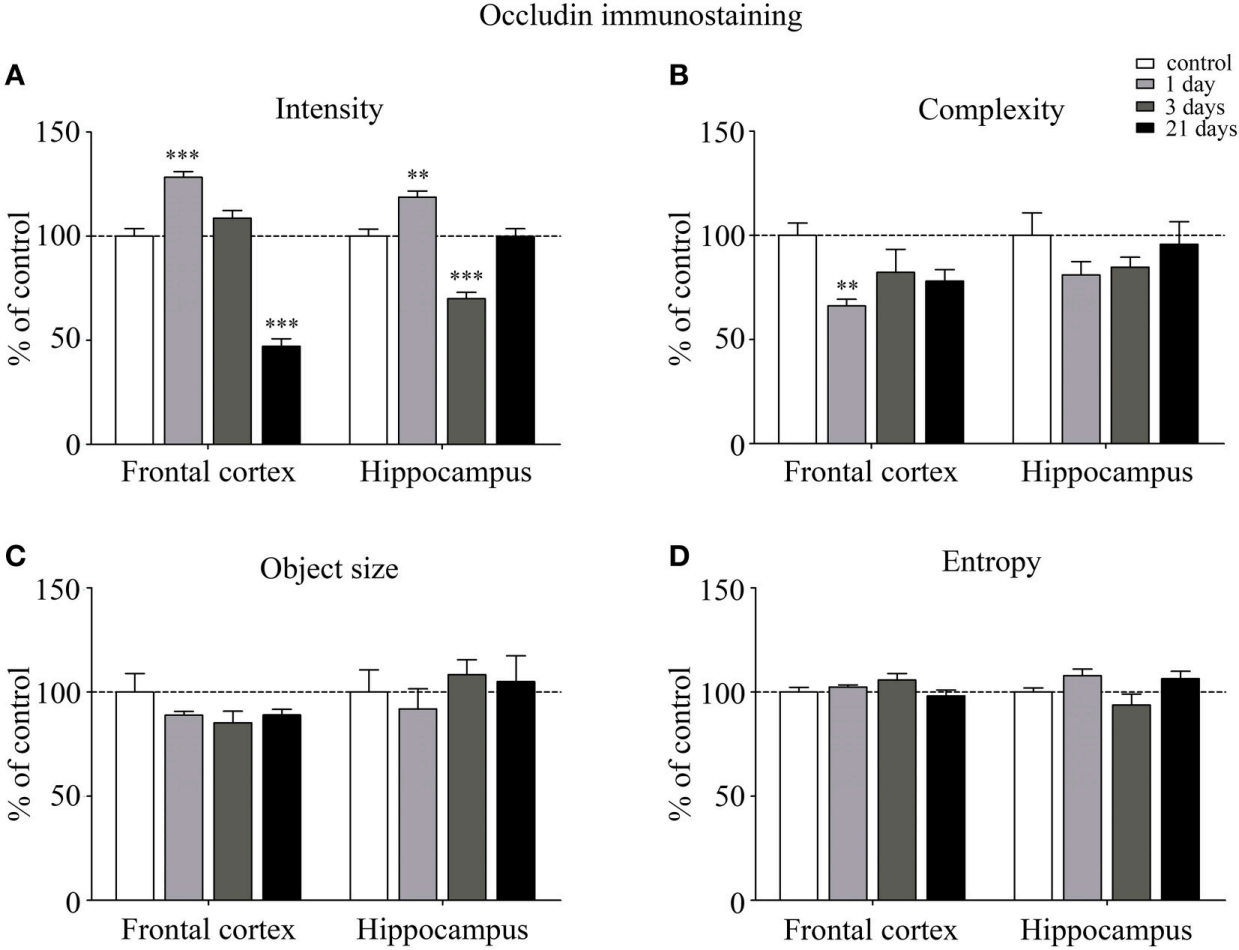
**Analysis of occludin immunofluorescence pattern in the frontal cortex and hippocampus of rats following restraint stress**. Graphs represent quantification of the intensity **(A)**, complexity **(B)**, mean object size **(C)** and entropy **(D)** of immunostained samples. Results are expressed as percentages of the unstressed control group. Values for each group are means ± SEM. Data were analyzed by One-way ANOVA followed by a Bonferroni *post hoc* test. ^**^p < 0.01, ^***^p < 0.001, significant differences as compared to the unstressed control group.

Occludin, the other integral membrane TJ protein analyzed in our sections, showed a significant increase (*p* < 0.001) in fluorescence intensity in both brain regions examined following 1-day stress, compared to the control group (Figure 5A). Contrary to this initial increase, on day 21 of restraint stress the fluorescence intensity of occludin was significantly reduced (*p* < 0.001) in the frontal cortex. In the hippocampus, a similar reduction in occludin fluorescence intensity was detected on day 3 of stress (Figure 5A). A significant decrease was observed in the complexity of occludin immunostaining in the frontal cortex after 1 day of stress (Figure 5B). The discontinuity of the occludin staining was visible in the stress groups in both regions. No change was seen for object size and entropy analyses (Figures 5C,D).

#### Immunostaining pattern of glucose transporter 1 (GLUT-1)

**Figure 6:**
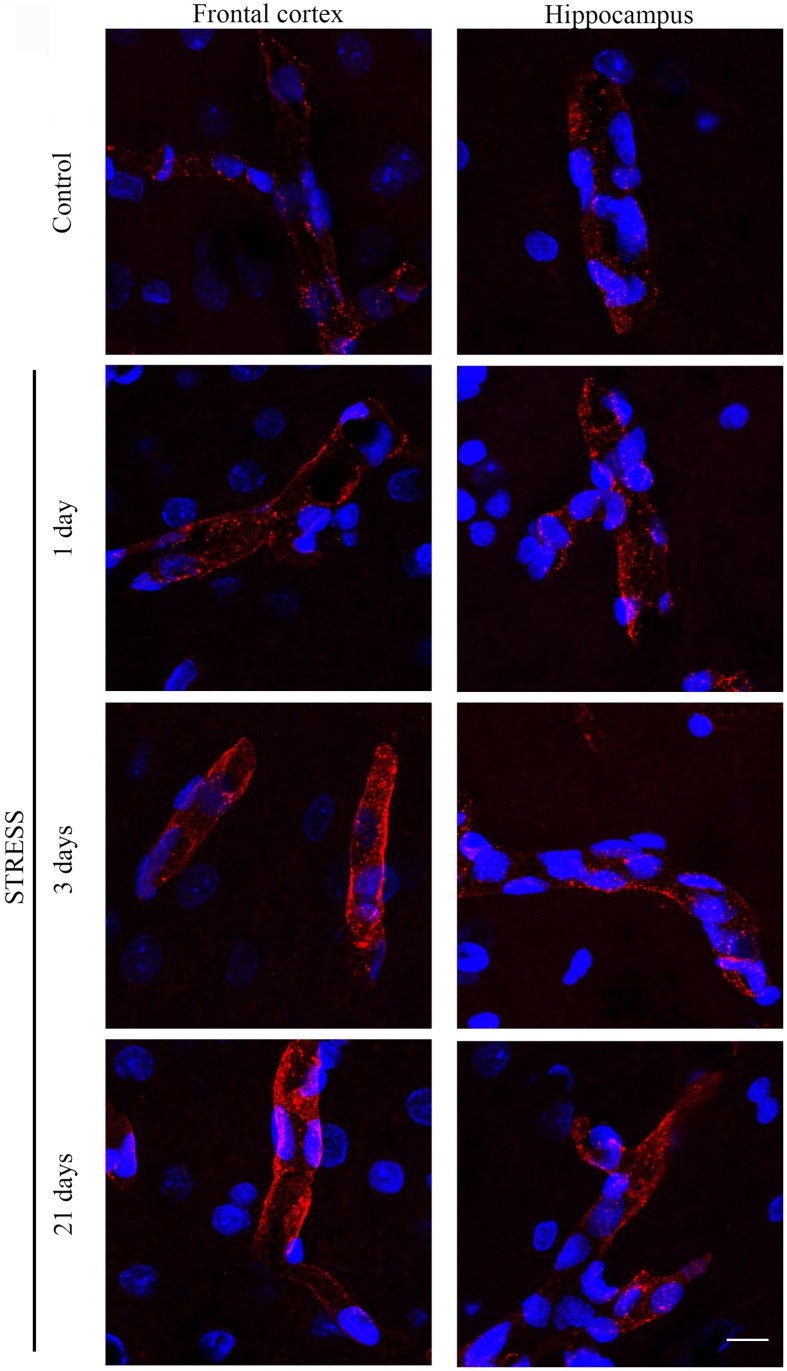
**Effect of restraint stress on GLUT-1 immunostaining of brain capillaries in the rat frontal cortex and hippocampus**. Glucose transporter-1 receptor was immunostained on brain sections of control and stressed rats. Red color: immunostaining for GLUT-1. Blue color: cell nuclei stained with Hoechst dye 33342. Scale bar: 15 μm.

**Figure 7:**
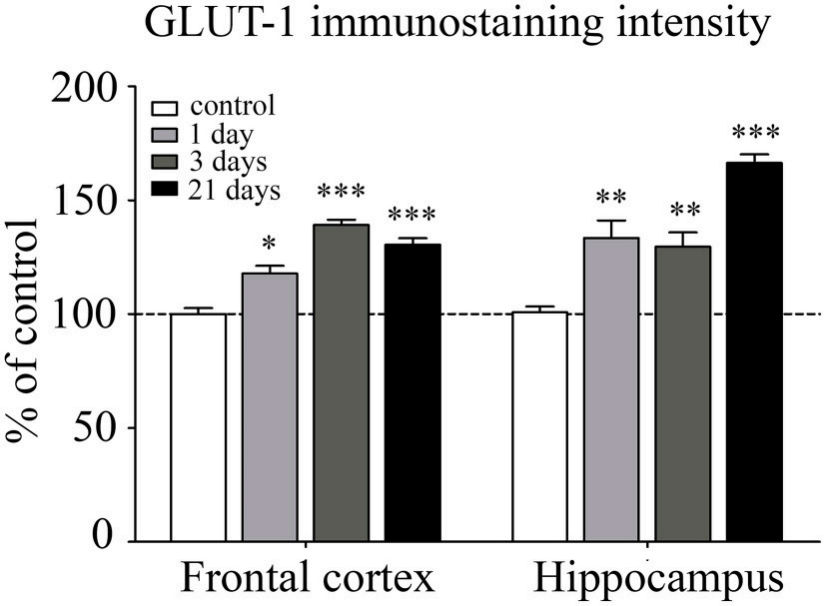
**Analysis of GLUT-1 immunofluorescence pattern in the frontal cortex and hippocampus of rats following restraint stress**. The graph represents the fluorescence intensity of the immunostaining. Results are expressed as percentages of the unstressed control group. Values for each group are means ± SEM. Data were analyzed by One-way ANOVA followed by Kruskal–Wallis test and Dunn's multiple comparison tests. ^*^p < 0.05, ^**^p < 0.01 and ^***^p < 0.001, significant differences as compared to the unstressed control group.

GLUT-1 is a BBB-specific glucose transporter and GLUT-1 immunostaining was observed only in brain endothelial cells. The effect of restraint stress on GLUT-1 immunostaining pattern of brain endothelial cells is shown on Figure 6. Fluorescence intensity of the GLUT-1 staining significantly increased in all restraint stress groups in the frontal cortex [day 1 (*p* < 0.05), day 3 (*p* < 0.001), and day 21 (*p* < 0.001)], as well as in the hippocampus [day 1 (*p* < 0.01), day 3 (*p* < 0.01), and day 21 (*p* < 0.001] compared to the unstressed control group (Figure 7).

#### Immunohistochemical analysis of astroglial endfeet

**Figure 8:**
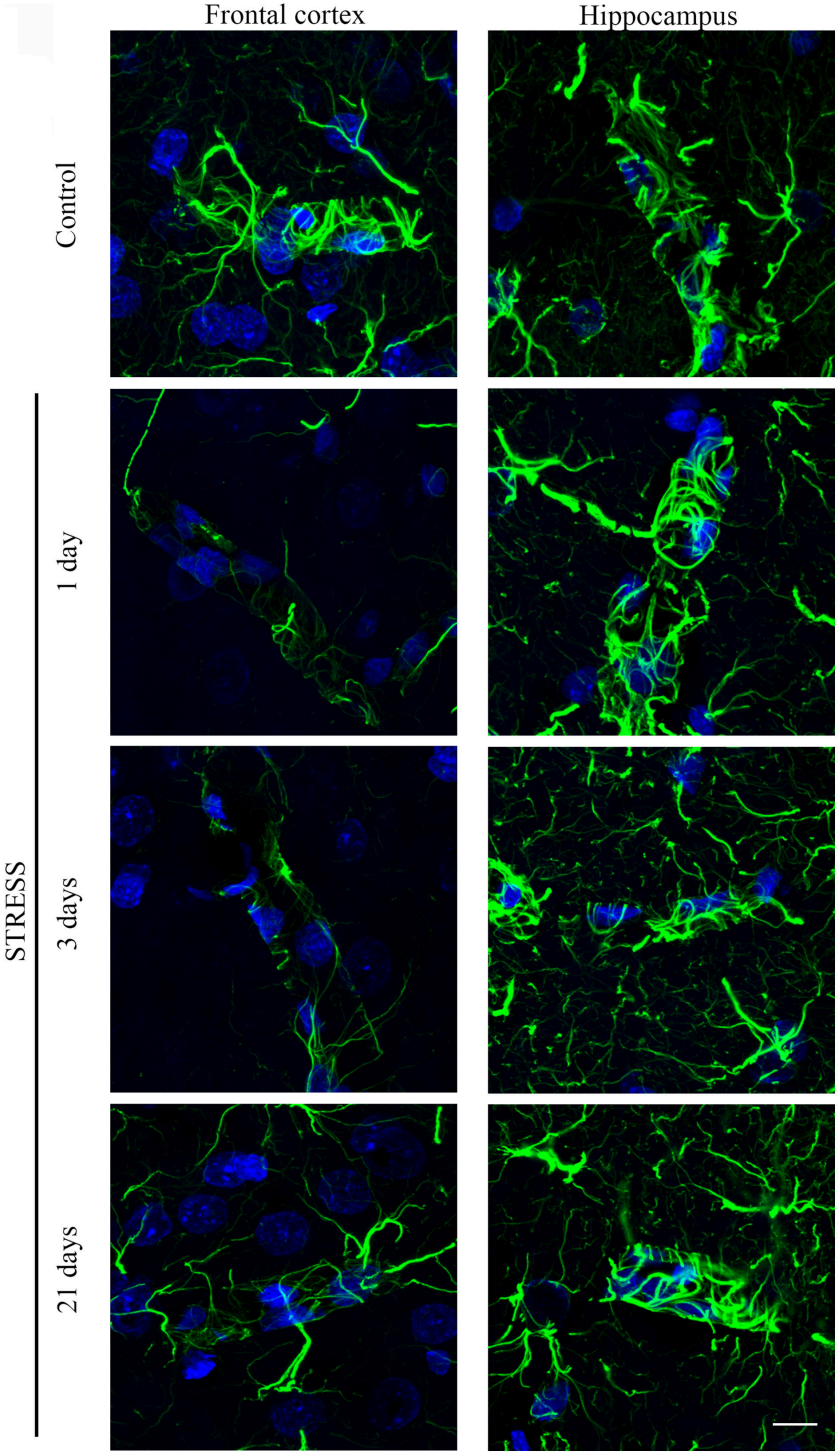
**Effect of restraint stress on GFAP immunostaining associated with brain capillaries in the rat frontal cortex and hippocampus**. GFAP was immunostained on brain sections of control and stressed rats. Green color: immunostaining for GFAP. Blue color: cell nuclei stained with Hoechst dye 33342. Scale bar: 15 μm.

**Figure 9:**
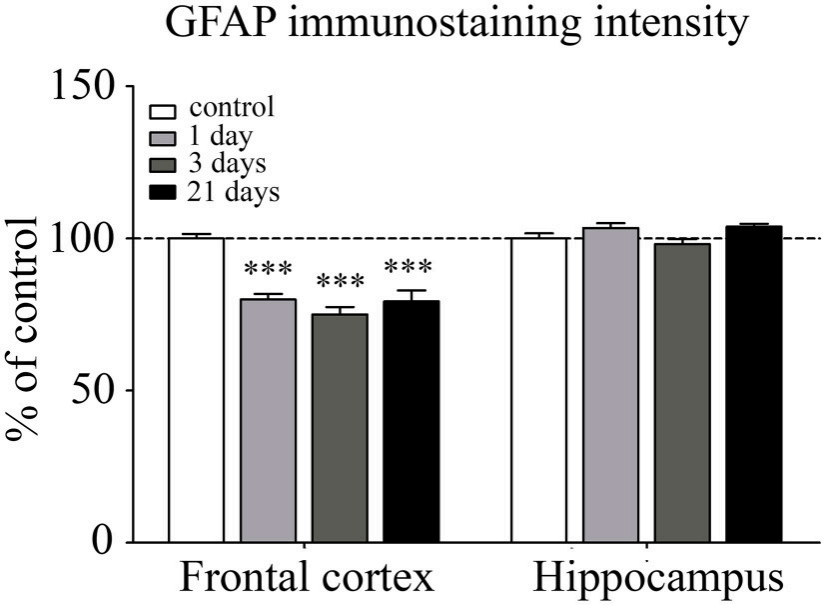
**Analysis of GFAP immunofluorescence pattern associated with capillaries in the frontal cortex and hippocampus of rats following restraint stress**. The graph represents the fluorescence intensity of the immunostaining. Results are expressed as percentages of the unstressed control group. Values for each group are means ± SEM. Data were analyzed by One-way ANOVA followed by Kruskal–Wallis test and Dunn's multiple comparison tests. ^***^p < 0.001, significant differences as compared to the unstressed control group.

Astroglial endfeet surrounding cerebral capillaries were visualized with GFAP immunohistochemistry (Figure 8). Following restraint stress, significant reductions in GFAP fluorescence intensity were observed in the frontal cortex. In contrast, restraint stress did not result in significant change in the GFAP staining intensity in the hippocampus at any time point tested (Figure 9).

#### Immunolabeling of neurons

**Figure 10:**
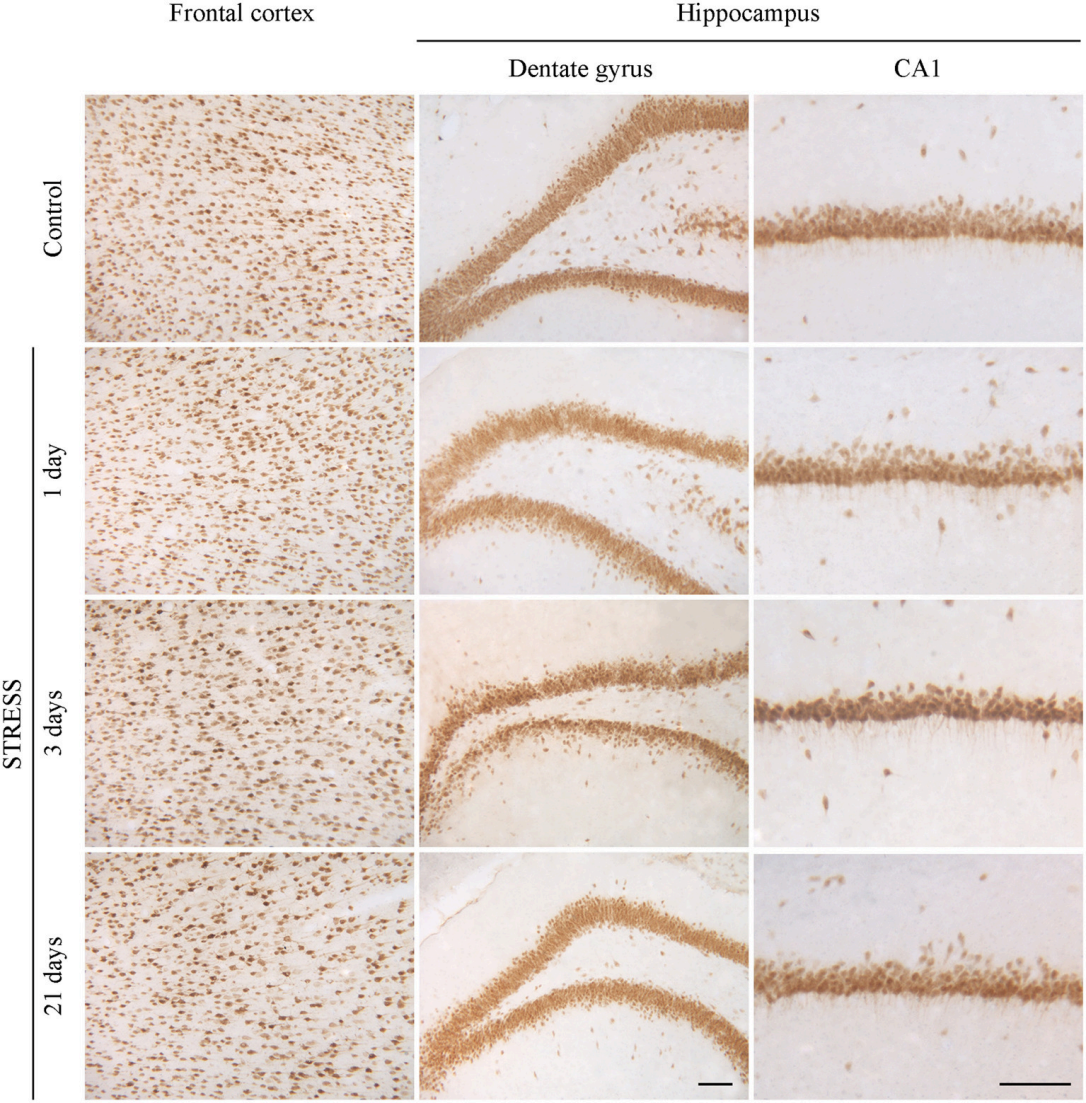
**Effect of restraint stress on NeuN immunoreactivity in the frontal cortex and hippocampus of rats**. The NeuN immunostaining (brown color) revealed neuronal cell bodies and nuclei on brain sections of control and stressed rats. Scale bar: 100 μm.

**Figure 11:**
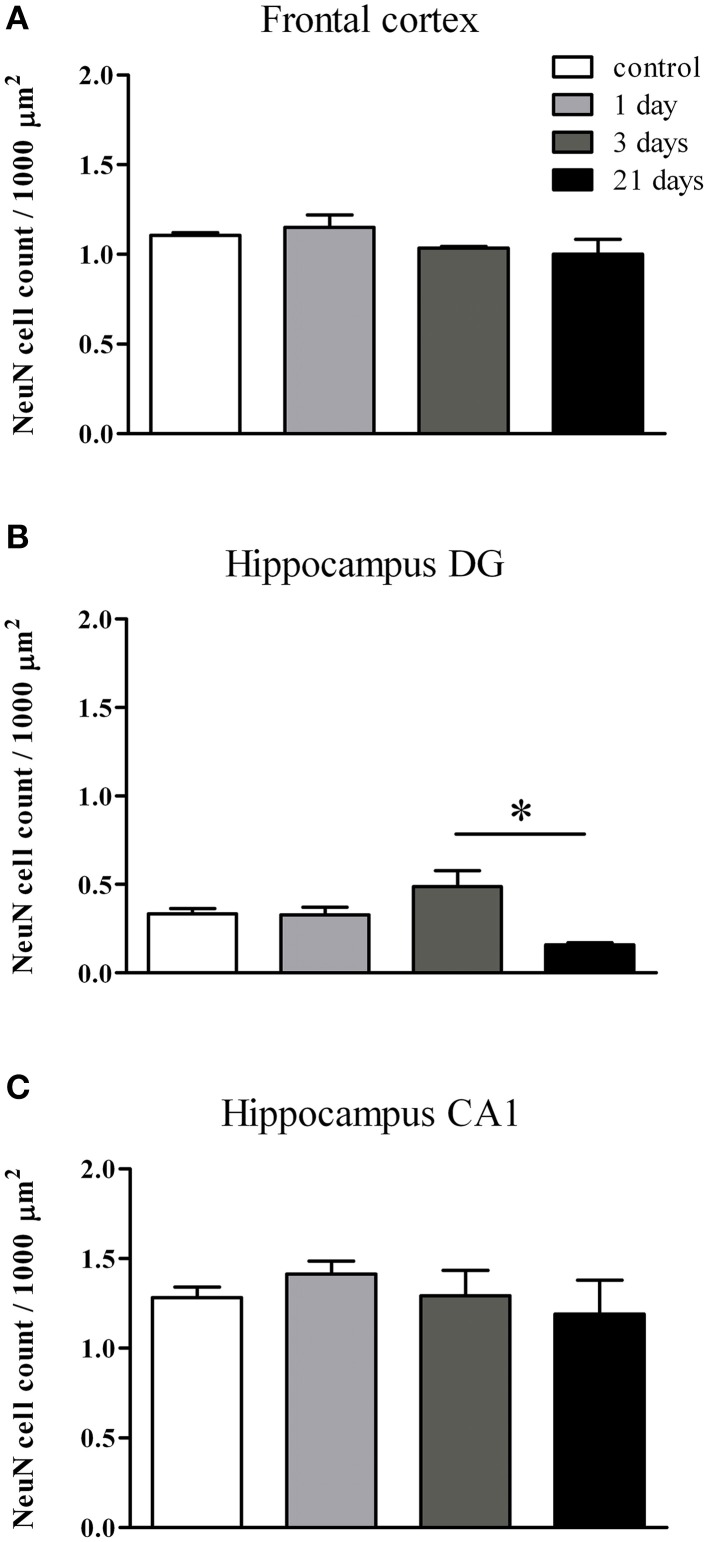
**Quantification of NeuN immunoreactive cells in the frontal cortex and hippocampus of rats following restraint stress**. The graphs represent the densities of NeuN expressing cells: frontal cortex **(A)**, dentate gyrus of hippocampus **(B)**, CA1 region of hippocmapus **(C)**. Values for each group are means ± SEM. Data were analyzed by One-way ANOVA followed by Kruskal–Wallis test and Dunn's multiple comparison tests. ^*^p < 0.05, significant difference as compared with the 3-day-stress group.

Neurons were immunostained using the neuronal marker NeuN. In the frontal cortex and in the CA1 region of the hippocampus both neuronal cell bodies and nuclei were immunoreactive for NeuN, while in the hippocampal dentate gyrus mostly neuronal cell bodies were labeled (Figure 10). Following restraint stress no significant changes in NeuN immunoreactive cell density were detected in the frontal cortex and the CA1 region of the hippocampus. A decreasing tendency in NeuN cell density was observed in the polymorphic region of the dentate gyrus after 21 days of stress compared with controls, which was significant compared to the density of NeuN expressing cells detected after 3 days of stress (Figure 11).

### Electron microscopy of section of brain capillaries

**Figure 12:**
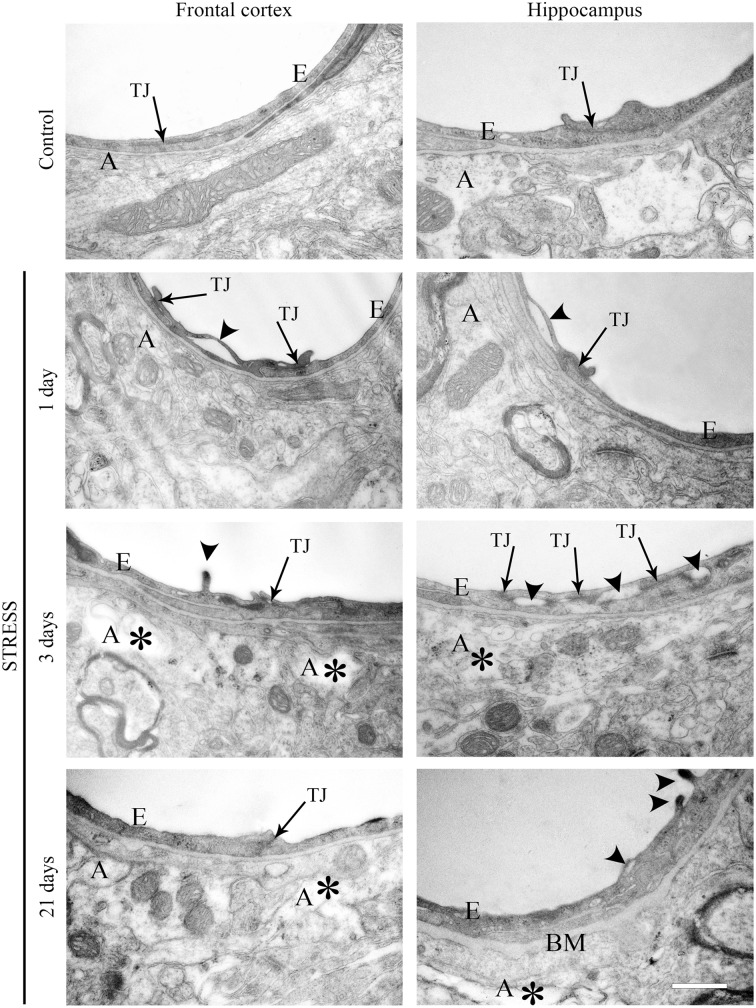
**Electron micrographs of brain capillaries in the frontal cortex and hippocampus of control and stressed rats**. E, brain endothelial cell; A, astrocyte; TJ, tight junction; BM, basal membrane; arrows indicate tight junctions; arrowheads point to brain endothelial deformities; asterisk marks edema in astrocytes. Scale bar: 500 nm.

Ultrastructural changes were detected in capillaries of the frontal cortex and hippocampus of stressed animals compared to controls (Figure 12). In control conditions thin brain endothelial cells with a smooth luminal membrane were observed. The contacting points of brain endothelial cells contained thick electrondense material, indicating tight intercellular junctions in the control groups (Table 1). The basal membrane was thin and connected to processes of fibrous astrocytes at the abluminal side. In rats after 1-day stress endothelial cells displayed morphological deformities: intraluminal protrusions, and partial detachments of endothelial cells from the basal membrane in both the frontal cortex and hippocampus were detected. The morphology of TJs was greatly changed, the number of discontinuous or open junctions increased. In the hippocampus edema of astrocytic processes was observed (Table 1). Three days of restraint stress resulted in more pronounced ultrastructural changes in both brain capillary endothelial cells and in astrocytes from the two regions. The majority of TJs was damaged and luminal protrusions were seen. Edematous changes in astroglial processes were characteristic in both brain regions examined. In animals which underwent stress for 21 days, capillary endothelial cells showed normal morphology in the frontal cortex, while in the hippocampus the number of open or discontinuous junctions was still higher. Furthermore, capillaries in both regions had basal membranes with increased thickness after 21 days of stress. Swollen astrocytes were present in the hippocampus, as well as in the frontal cortex in this experimental group (Table 1).

**Table 1:** **Summary of changes in BBB ultrastructure following restraint stress in adult rats**.

			**Control**	**Stress**		
				**1 day**	**3 days**	**21 days**
**FRONTAL CORTEX**						
Number of capillaries			13	15	11	18
Number of images			33	49	82	100
Brain capillary endothelial cell	Luminal membrane	Smooth	91%	65%	68%	91%
		Protrusions	9%	35%	32%	6%
	Tight junctions	Intact	62%	0%	22%	58%
		Discontinuous	38%	100%	78%	42%
	Basal membrane	Intact	88%	88%	84%	56%
		Increased thickness	12%	12%	16%	44%
		Detachment	0%	4%	4%	8%
Astroglia endfeet		Intact	94%	94%	34%	19%
		Edematous	6%	6%	66%	81%
**HIPPOCAMPUS**						
Number of capillaries			19	14	10	15
Number of images			85	56	46	82
Brain capillary endothelial cell	Luminal membrane	Smooth	77%	61%	85%	78%
		Protrusions	23%	39%	15%	22%
	Tight junctions	Intact	94%	28%	25%	50%
		Discontinuous	6%	72%	75%	50%
	Basal membrane	Intact	87%	84%	85%	39%
		Increased thickness	13%	16%	15%	61%
		Detachment	0%	5%	6%	5%
Astroglia endfeet		Intact	92%	77%	50%	30%
		Edematous	8%	23%	50%	70%

*Values are shown as percent of all images analyzed in the group, except for tight junctions, which are given as percent of all junctions observed in the group*.

## Discussion

Stress stimuli, including immobilization stress, are reported to induce neurological symptoms that are related to BBB dysfunctions (Sharma and Dey, 1988; Abdel-Rahman et al., 2002; Sharma et al., 2012). Previous studies prove that stress increases BBB permeability in rodents (Sharma et al., 1991; Foti Cuzzola et al., 2013) and in humans (Hanin, 1996), which is linked to disturbances of CNS homeostasis and neuronal death (Deli, 2005; Zlokovic, 2008). Although there are studies describing stress-induced BBB alterations, information on stress-related morphological changes at the BBB is very limited. Therefore, this is the first study which demonstrates morphological changes at light and electron microscopic level in brain endothelial cells and astroglial endfeet following restraint stress at three different time points in rats (Table 2).

**Table 2:** **Summary of the changes in immunostaining for BBB markers and ultrastructural alterations of the BBB following restraint stress in rats**.

		**Frontal Cortex**			**Hippocampus**		
		**1 day**	**3 days**	**21 days**	**1 day**	**3 days**	**21 days**
**BRAIN ENDOTHELIAL CELLS**							
**Immunostainings**							
Claudin-5	Intensity	−	−	−	↓	−	−
	Complexity	↓	↓	↓	↓	↓	↓
	Entropy	−	−	↑	−	−	↑
Occludin	Intensity	↑	−	↓	↑	↓	−
	Complexity	↓	−	−	−	−	−
	Entropy	−	−	−	−	−	−
GLUT-1	Intensity	↑	↑	↑	↑	↑	↑
**Electron Microscopy**							
	TJ integrity	↓	↓	−	↓	↓	↓
	BM integrity	−	−	↓	−	−	↓
**ASTROCYTES**							
**Immunostainings**							
GFAP	Intensity	↓	↓	↓	−	−	−
**Electron Microscopy**							
	Edema	−	+	+	+	+	+

*BM, basal membrane; GFAP, glial fibrillary acidic protein; GLUT-1, glucose transporter-1; TJ, tight junction; −, no change; ↓, decrease; ↑, increase; +, edema observed*.

TJs play a key role in establishing the barrier function of brain endothelial cells (Abbott et al., 2010). Our present results demonstrate alterations in the morphology of the most expressed brain endothelial TJ structural proteins claudin-5 and occludin. The pattern of the junctional networks was analyzed by measuring the entropy and complexity of the immunostained structures for the first time. An extended network of branched interendothelial junctions was revealed by claudin-5 and occludin immunostaining in brain microvessels, similarly to staining patterns described in microvessels from mouse brains (Song and Pachter, 2004) and in human adult cortex (Virgintino et al., 2004). Analysis of claudin-5 immunostained samples indicates an increase in entropy in both the frontal cortex and hippocampus at 21 days of stress. The concept of entropy originally comes from physics, where it is used to describe the randomness of a thermodynamic system (Clausius, 1850). By analogy, Shannon (1948) introduced a formally similar quantity in information theory to describe the unpredictability of information content of messages. This notion can be applied in image analysis, as well, in order to quantify the orderliness of a picture (Gonzalez et al., 2003). Entropy is especially useful when monitoring changes in the highly organized biological tissue images, such as those of the brain endothelial tight junctions. Here, the loss, fragmentation, reorganization or redistribution of the immunostaining by injury is expected to yield an increased entropy of the corresponding images. A special advantage of the concept is that it does not require any special assumption to identify vessels, but, in this context, it is a model-independent approach. Moreover, the complexity of claudin-5 protein was significantly reduced in all stress groups in both regions examined, indicating that the claudin-5 staining pattern in the frontal cortex and hippocampus displays less branching points in stressed animals, compared to the control group. Notably, the immunoreactivity pattern of occludin, the other TJ protein studied, revealed a similar reduction in complexity only in response to 1-day stress, which could be detected in the frontal cortex. Thus, the pattern of the two TJ proteins examined does not change in parallel, and it seems that claudin-5 staining is more sensitive to stress conditions. There are two studies indicating, that indeed, claudin-5 and occludin levels or staining patterns may not change parallelly in pathologies. Chronic (10-day) unpredictable stress in rats significantly reduced the level of claudin-5, but not that of occludin in isolated brain microvessels (Northrop and Yamamoto, 2012). In experimental autoimmune encephalomyelitis, a well-characterized model of multiple sclerosis changes in claudin-5 immunostaining in brain vessels were evident at a very early disease stage, while the initially preserved occludin pattern showed alterations at more advanced stages (Errede et al., 2012).

Quantitative changes in the immunostaining of TJ proteins were analyzed based on fluorescence intensity measurements. Differences were revealed between changes in fluorescence intensity and localization pattern of the same protein. While the entropy and complexity analysis of claudin-5 immunoreactivity showed alterations in the localization of this protein in stressed animals, the fluorescence intensity of claudin-5 did not change with the exception of the hippocampus in animals suffering 1-day stress. Occludin response to stress, in contrast, was manifested in fluorescence intensity changes rather than localization: an increase in occludin fluorescence intensity was detected after acute stress, followed by a decrease in animals suffering chronic stress. Recently, a morphological study performed in a mouse acute immobilization stress model demonstrated extravasation of Evan's blue dye to brain tissue, and found no claudin-1, claudin-3, and ZO-1 immunoreactivity in brain vessels using paraffin sections and classical peroxidase diaminobenzidine technique (Foti Cuzzola et al., 2013). Although this study examined three important TJ proteins, the immunostaining pattern of claudin-5 and occludin was not studied. Similarly, restraint stress induced ultrastructural changes in endothelial cells have not been described before in the frontal cortex and hippocampus. Our electron microscopical observations demonstrated morphological changes of brain endothelial cells in all stressed groups. The disruption of TJs and detachment of brain endothelial cells from the capillary basal membrane with alterations in the immunostaining pattern of claudin-5 and occludin suggest stress induced BBB dysfunction. In several models of neurological diseases, especially stroke or cerebral ischemia-reperfusion injury and Alzheimer's disease a link was established between decreased expression, disrupted organization or redistribution of claudin-5 and occludin and increased BBB permeability (Willis et al., 2010; Jiao et al., 2011; Yang and Rosenberg, 2011; Hartz et al., 2012). While in the present study functional BBB permeability was not examined, similar morphological changes were described as an indirect proof of BBB opening.

Glucose transport across the BBB is essential to provide the brain with energy and maintain its metabolic function. GLUT-1 is highly expressed in brain endothelial cells and has a crucial role in providing glucose for the CNS (Zlokovic, 2008). Since glucose uptake correlates with GLUT-1 levels, quantitative changes in the GLUT-1 protein may suggest altered glucose metabolism of the brain linked to the development of cognitive impairment and movement disorders (Winkler et al., 2015). In our experimental model restraint stress significantly increased the fluorescence intensity of GLUT-1 labeling in the frontal cortex and hippocampus in rats. There is no consensus in the literature regarding GLUT-1 expressional changes in neurodegenerative and stress conditions. A decreased expression and activity of GLUT-1 were described at the BBB of AD patients (Horwood and Davies, 1994; Erickson and Banks, 2013) and in aged transgenic mice used as animal models of AD (Hooijmans et al., 2007; Winkler et al., 2015). In contrast, prenatal stress causes elevation in the GLUT-1 expression in the rat frontal cortex and hippocampus (Detka et al., 2014). Mixed modality chronic stress paradigm lasting for 11 days and including six sessions of restraint stress increased GLUT-1 gene expression in the hippocampus of adult male rats (Kelly et al., 2014). Our results are in accordance with these latter data suggesting an increase in the glucose demand of the brain following stress stimuli and elevated GLUT-1 expression as a short-term compensatory mechanism.

Stress may have an impact on astroglial endfeet too, since astrocytes are key participants in inducing and maintaining BBB properties in endothelial cells (Abbott, 2002; Abbott et al., 2006). Furthermore, either upregulation or downregulation of GFAP results in BBB disruption *in vivo* (Willis, 2012), and GFAP deficient astrocytes fail to induce BBB properties in co-culture (Pekny et al., 1998). In our study a significant decrease in GFAP fluorescence intensity was found in the frontal cortex in astroglial endfeet associated with capillaries following restraint stress. In contrast, in the hippocampus no change in GFAP content was seen around microvessels in stressed animals compared to controls. There is a well-known regional heterogeneity of astrocytic morphology and function (Emsley and Macklis, 2006; Hewett, 2009; Oberheim et al., 2012). The density of GFAP astrocytes is significantly higher, while the proliferation rate is lower in the hippocampus than in the cortex of adult mice (Emsley and Macklis, 2006) which may explain why the decrease in fluorescence intensity of GFAP staining is detectable in the frontal cortex, but not in the hippocampus in our study. Data published in the literature are contradictory regarding restraint stress induced alterations in GFAP content of different brain areas. The observed reduction in GFAP content due to stress stimuli in the frontal cortex is in accordance with results on GFAP immunoreactivity and concentration in the periaqueductal gray matter in rats suffering subacute and chronic restraint stress (Imbe et al., 2012). In Sprague–Dawley rats repeated immobilization stress lasting for 3 weeks resulted in an increase in GFAP expression in both the hippocampus and cortex (Jang et al., 2008), while no change in GFAP concentration was reported in protein samples from isolated cortical microvessels following chronic unpredictable stress (Northrop and Yamamoto, 2012). The controversies among data may result, at least in part, from the difference in the type of stress, strain of rats and the experimental methods applied. In addition, our study focused on the fluorescence intensity of GFAP in microvessel-associated glial endfeet within a particular brain area and did not examine global GFAP immunoreactivity in the selected two brain regions. Electron microscopic analysis of perivascular astroglial cells revealed edematous astrocytic processes in both the frontal cortex and hippocampus of rats following restraint stress lasting for 3 or 21 days. These results are in line with earlier studies; swelling of astrocytes is often associated with BBB disruption in the cerebral cortex (Gajkowska et al., 2011; Ezan et al., 2012). Moreover, swollen astrocytic endfeet are also reported in pigs susceptible to stress (Vandenhaute et al., 2012). The present study suggests that restraint stress leads to astrocytic swelling which becomes prominent at the third day and it persists at least till 21 days of stress. These structural alterations of astrocytes may represent morphological signs of BBB dysfunction.

NeuN is a soluble nuclear protein and a marker for mature neurons the expression of which is sensitive to injury (Unal-Cevik et al., 2004; Argandoña et al., 2012). Unal-Cevik et al. (2004) found that a significant number of neurons lose their NeuN positivity after cerebral ischemia, but preserve their integrity. In another study loss of NeuN staining was an early marker of injured, but still rescuable cortical neurons (Argandoña et al., 2012). In our study restraint stress did not cause change in neuronal cell density in the frontal cortex and in the CA1 region of hippocampus, while a trend for a decreased density was observed in the dentate gyrus of hippocampus, which is particularly sensitive to stress (Zitman and Richter-Levin, 2013). These findings indicate, that the morphological changes at the level of BBB caused by restraint stress with a duration between 1 day and 3 weeks described in the present study do not result in neuronal damage. However, chronic 8-week stress in mice caused neuronal damage in the same brain regions (Huang et al., 2015) suggesting time-dependent effect of stress.

In conclusion, our study provides evidence on alterations in immunolabeling of key proteins of the BBB following restraint stress. Furthermore, our observations demonstrate that the ultrastructure of brain endothelial cells and astroglial endfeet is modified in stress conditions. Our morphological findings may help to explain the stress induced BBB functional changes playing a role in the pathogenesis of several neurological and psychiatric diseases.

## Author contributions

Conceived and designed the experiments: MD, SV, PS, MP, JK. Performed the experiments: PS, SV, ZH, FRW, A Kittel, AET, LK, MM, ZO. Analyzed the data: PS, ZH, MM, A Kincses, A Kittel, GS, AD. Contributed reagents/materials/analysis tools: MD, MP, JK, GS, AD, GR. Wrote the paper: PS, ZH, MD, A Kittel, AD.

### Conflict of interest statement

The authors declare that the research was conducted in the absence of any commercial or financial relationships that could be construed as a potential conflict of interest.
